# Alginate Oligosaccharide Prevents Acute Doxorubicin Cardiotoxicity by Suppressing Oxidative Stress and Endoplasmic Reticulum-Mediated Apoptosis

**DOI:** 10.3390/md14120231

**Published:** 2016-12-20

**Authors:** Jun-Jie Guo, Lei-Lei Ma, Hong-Tao Shi, Jian-Bing Zhu, Jian Wu, Zhi-Wen Ding, Yi An, Yun-Zeng Zou, Jun-Bo Ge

**Affiliations:** 1 Department of Cardiology, Shanghai Institute of Cardiovascular Diseases, Zhongshan Hospital, Fudan University, 180 Feng Lin Road, Shanghai 200032, China; qy_junjie@163.com (J.-J.G.); mllsdjn@126.com (L.-L.M.); hongtaoshi@126.com (H.-T.S.); zhujianbing543@163.com (J.-B.Z.); everwoo@sina.com (J.W.); zhiwen.d@gmail.com (Z.-W.D.); 2 Department of Cardiology, The Affiliated Hospital of Qingdao University, 16 Jiang Su Road, Qingdao 266003, China; any@medmail.com.cn; 3 Institute of Biomedical Science, Fudan University, Shanghai 200032, China

**Keywords:** alginate oligosaccharide, doxorubicin cardiotoxicity, oxidative stress, endoplasmic reticulum, apoptosis

## Abstract

Doxorubicin (DOX) is a highly potent chemotherapeutic agent, but its usage is limited by dose-dependent cardiotoxicity. DOX-induced cardiotoxicity involves increased oxidative stress and activated endoplasmic reticulum-mediated apoptosis. Alginate oligosaccharide (AOS) is a non-immunogenic, non-toxic and biodegradable polymer, with anti-oxidative, anti-inflammatory and anti-endoplasmic reticulum stress properties. The present study examined whether AOS pretreatment could protect against acute DOX cardiotoxicity, and the underlying mechanisms focused on oxidative stress and endoplasmic reticulum-mediated apoptosis. We found that AOS pretreatment markedly increased the survival rate of mice insulted with DOX, improved DOX-induced cardiac dysfunction and attenuated DOX-induced myocardial apoptosis. AOS pretreatment mitigated DOX-induced cardiac oxidative stress, as shown by the decreased expressions of gp91 (phox) and 4-hydroxynonenal (4-HNE). Moreover, AOS pretreatment significantly decreased the expression of Caspase-12, C/EBP homologous protein (CHOP) (markers for endoplasmic reticulum-mediated apoptosis) and Bax (a downstream molecule of CHOP), while up-regulating the expression of anti-apoptotic protein Bcl-2. Taken together, these findings identify AOS as a potent compound that prevents acute DOX cardiotoxicity, at least in part, by suppression of oxidative stress and endoplasmic reticulum-mediated apoptosis.

## 1. Introduction

Doxorubicin (DOX), an anthracycline antibiotic, is a widely used and highly potent chemotherapeutic agent to treat a broad range of cancers. However, the clinical usage of DOX is greatly limited by its dose-dependent and time-dependent cardiotoxicity [1]. The mechanisms of DOX-induced cardiotoxicity have not been fully elucidated. A plethora of deleterious effects such as excessive reactive oxygen species (ROS) generation, lipid peroxidation, DNA/RNA damage, calcium mishandling, inhibition of autophagic flux and endoplasmic reticulum-mediated apoptosis have all been linked to DOX-induced cardiotoxicity [2,3,4]. Anti-oxidative therapies including using antioxidants and transgenic mice overexpressing anti-oxidative enzymes have been verified to be effective in protecting against DOX-induced cardiotoxicity [5,6,7,8]. A recent study has demonstrated that a chemical endoplasmic reticulum chaperone could obliterate DOX-induced cardiac dysfunction [9]. These observations hint that some agents may alleviate DOX-induced cardiotoxicity by inhibiting oxidative stress or endoplasmic reticulum-mediated apoptosis.

Alginate, an acidic polysaccharide consisting of alternative blocks of β-d-mannuronic acid (M) and α-l-guluronic acid (G), is extracted from various marine brown algae and has been widely used in various fields, such as food, tissue engineering, and drug delivery, because of its non-toxic, non-immunogenic characteristics [10]. Alginate oligosaccharide (AOS), produced by depolymerizing alginate using different degradation methods including enzymatic degradation, acid hydrolysis and oxidative degradation, has been proven to exert several pharmacological activities, including anti-oxidative [11], anti-apoptotic [12], anti-inflammatory [13] and anti-proliferative effects [14]. Nevertheless, whether AOS is protective against acute DOX cardiotoxicity is not yet clear, and the underlying mechanisms need to be elucidated. For this purpose, the present study aimed to evaluate the protective effect of AOS acquired by an enzymatic degradation method on acute DOX cardiotoxicity. To explore the potential mechanisms of this protection, we examined the influences of AOS on the oxidative stress and endoplasmic reticulum-mediated apoptosis.

## 2. Results

### 2.1. AOS Pretreatment Preserves DOX-Induced Cardiac Dysfunction in Mice

**Figure 1:**
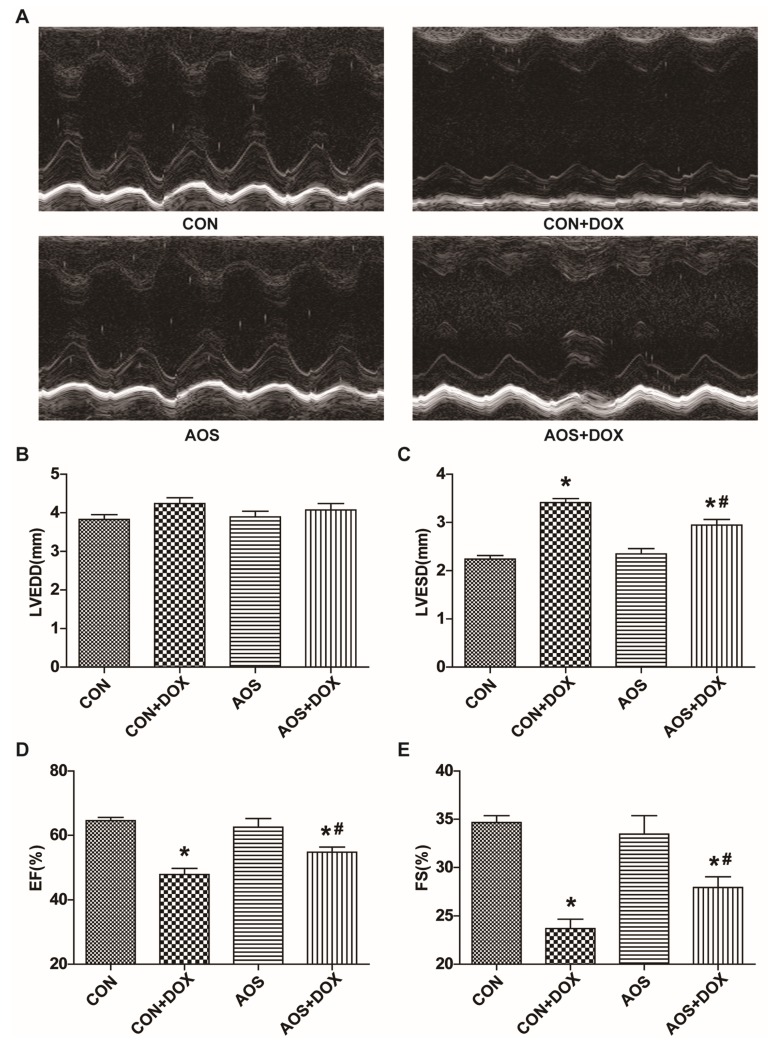
AOS pretreatment attenuates doxorubicin (DOX)-induced cardiac contractile dysfunction. (**A**) Cardiac function of mice receiving DOX injection with or without AOS pretreatment (200 mg/kg/day, 7 days) was measured after five days, and representative echocardiographic images were acquired; (**B**–**E**) Cardiac function was measured after five days. LVEDD: left ventricular end-diastolic dimension; LVESD: left ventricular end-systolic dimension; EF: left ventricular ejection fraction; FS: left ventricular fractional shortening. * *p* < 0.05 vs. the control (CON) group; ^#^ *p* < 0.05 vs. the CON + DOX group, *n* = 6 in each group.

To evaluate the cardiac function, we used a dose of 15 mg/kg DOX because of the high morbidity induced by the dose of 20 mg/kg DOX. First, we observed whether AOS treatment (200 mg/kg/day, five days) after DOX injection could exert a protective effect on cardiac dysfunction induced by DOX insult. Unfortunately, no significant improvement was observed (Figure S5), and further research was not pursued. Then, the cardioprotection of AOS pretreatment (200 mg/kg/day, seven days) was observed. All mice remained alive on the fifth day after DOX administration, and representative echocardiograms are shown in Figure 1A. Compared with the control (CON) group, mice pre-treated with AOS alone did not exhibit abnormal cardiac function, and there was no difference in left ventricular end-diastolic dimensions (LVEDD) among the four groups (Figure 1B). Surprisingly, mice receiving a DOX injection exhibited a significant increase of 52% for left ventricular end systolic dimensions (LVESD) compared to the CON group, whereas this alteration was markedly restored in the AOS + DOX group (Figure 1C). Moreover, DOX insult induced a marked decrease of 37% and 30%, respectively, in cardiac contractility characterized by the left ventricular ejection fraction (EF) and fractional shortening (FS), and these effects were significantly attenuated by AOS pretreatment (Figure 1D,E).

### 2.2. AOS Pretreatment Attenuates Acute DOX-Induced Cardiotoxicity in Mice

**Figure 2:**
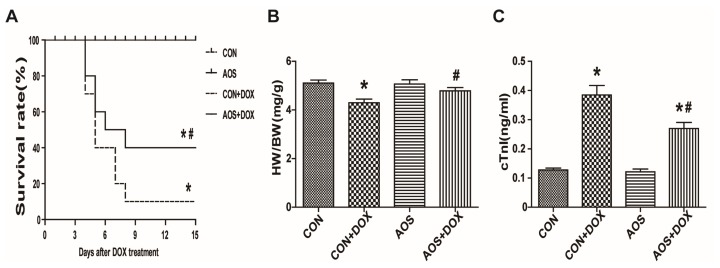
AOS pretreatment attenuates acute DOX cardiotoxicity in mice. (**A**) Kaplan-Meier survival curve analysis of mice after DOX injection with or without AOS pretreatment (200 mg/kg/day, *7* days). *n* = 10 in each group; (**B**) Graphs showing the heart weight to body weight ratio. HW: heart weight; BW: body weight. *n* = 6 in each group; (**C**) Evaluation of plasma cTnI levels. *n* = 6 in each group. * *p* < 0.05 vs. the CON group; ^#^ *p* < 0.05 vs. the CON + DOX group.

The general status of mice in the four groups was observed daily after the DOX insult. Mice in CON + DOX group appeared lethargic and weak with weight loss, and 90% of them had died by eight days after DOX (20 mg/kg) treatment (Figure 2A). However, these signs were attenuated in the mice pre-treated with AOS (200 mg/kg/day, seven days) and mortality was decreased to 40% by 15 days. The mice in both the CON and AOS groups were all alive over the entire observation period (15 days). The heart-to-body weight ratio (Figure 2B) on the fifth day after DOX injection (15 mg/kg) was obviously decreased compared with the CON group, but the AOS pretreatment (200 mg/kg/day, seven days) significantly improved the loss of the heart-to-body weight ratio compared with the CON + DOX group. Similarly, cTnI—a specific biomarker of cardiac injury—was significantly increased in the CON + DOX group compared with the CON and AOS groups. However, the cTnI level was significantly decreased in the AOS + DOX group compared with the CON + DOX group (Figure 2C).

### 2.3. AOS Pretreatment Protects DOX-Induced Myocardial Histological Alterations and Apoptosis in Mice

**Figure 3:**
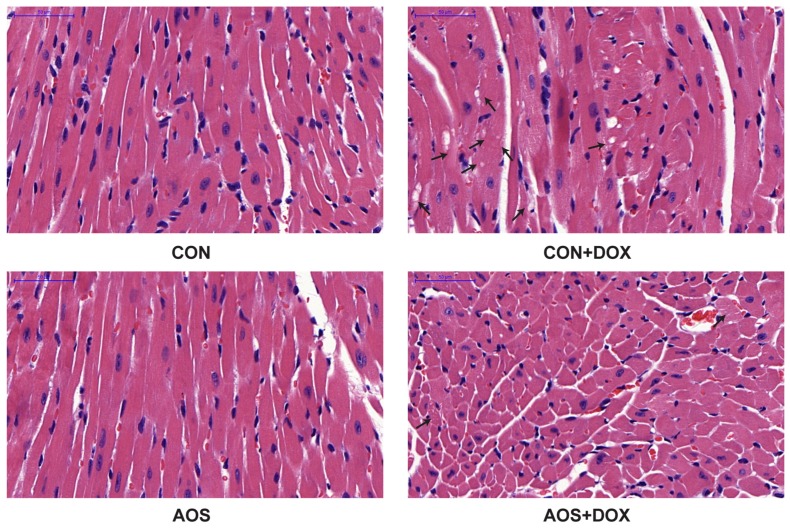
Effect of AOS pretreatment on DOX-induced myocardial histological alterations. Representative histopathological findings at 400× magnification of mouse hearts stained with hematoxylin and eosin (HE). Black arrows indicate extensive cytoplasmic vacuolization and nuclear condensation or dissolution, *n* = 6 in each group.

**Figure 4:**
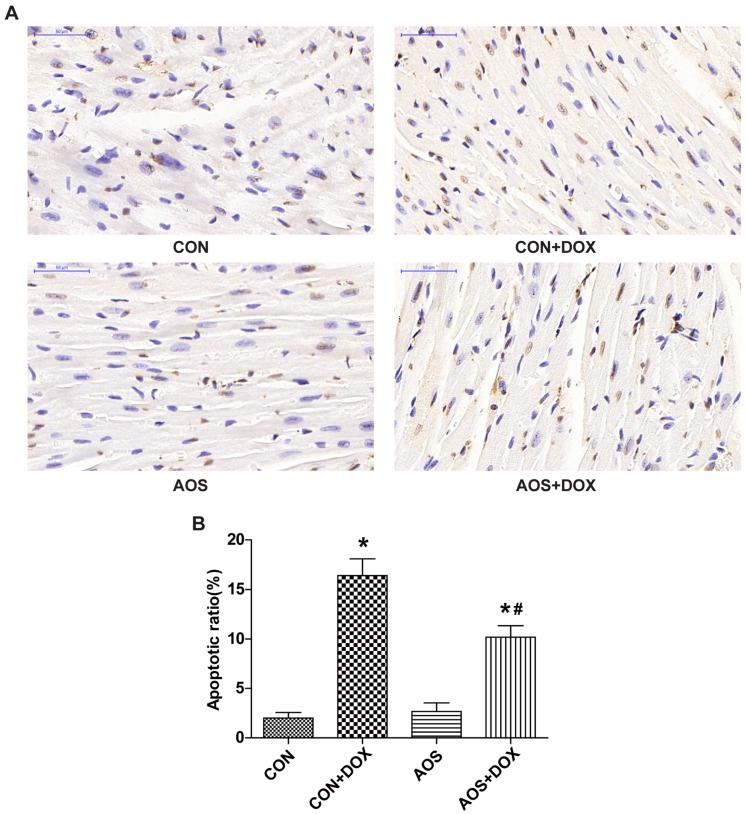
AOS pretreatment decreases DOX-induced myocardial apoptosis. (**A**) Representative photomicrographs at 400× magnification of mouse hearts stained with TUNEL, apoptotic cardiomyocyte nuclei appear brown-stained, whereas normal nuclei appear blue; (**B**) Quantitative analysis of the percentage of TUNEL-positive cells. * *p* < 0.05 vs. the CON group; ^#^ *p* < 0.05 vs. the CON + DOX group, *n* = 6 in each group.

To examine whether AOS pretreatment (200 mg/kg/day, seven days) attenuates DOX-induced cardiac injury, we analyzed the heart sections with hematoxylin and eosin (HE) staining through electron microscopy. As shown in Figure 3, AOS by itself had no effect on cardiac morphology. Consistent with a previous study [15], DOX-treated mice exhibited extensive focal cytoplasmic vacuolization, a specific change of DOX-induced cardiac injury, whereas this effect was significantly reduced by AOS pretreatment. Terminal deoxynucleotidyl transferase-mediated dUTP nick end labeling (TUNEL) assays were performed to determine the effect of AOS on apoptosis in DOX-treated hearts. As shown in Figure 4, TUNEL-positive cardiomyocytes were rarely observed in the heart sections of mice in the CON and AOS groups. Conversely, a significantly larger number of TUNEL-positive cardiomyocytes (16.6%) were detected in the CON + DOX group, whereas this change was significantly mitigated by AOS pretreatment (9.6%).

### 2.4. AOS Pretreatment Reduces DOX-Induced Oxidative Stress in Mice

**Figure 5:**
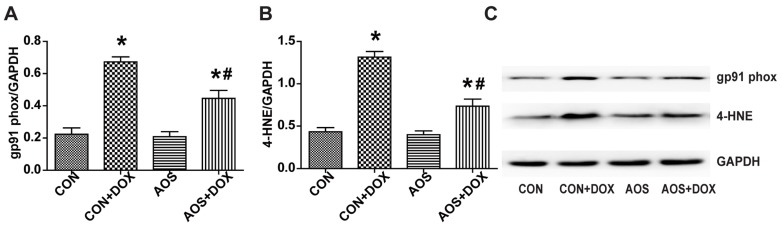
AOS pretreatment inhibits oxidative stress in the heart treated with DOX. (**A**,**B**) Quantitative analyses of gp91 phox and 4-HNE protein expression; (**C**) Representative blots of gp91 phox, 4-HNE and glyceraldehyde-3-phosphate dehydrogenase (GAPDH). * *p* < 0.05 vs. the CON group; ^#^ *p* < 0.05 vs. the CON + DOX group, *n* = 6 in each group.

Nicotinamide-adenine dinucleotide phosphate (NADPH) oxidases are involved in DOX-induced oxidative stress, and DOX-stimulated NADPH-dependent superoxide production in the heart is critically dependent on gp91 (phox) [8]; therefore, we measured the protein expression of gp91(phox) in the hearts. As shown in Figure 5A, AOS alone did not have an obvious effect on the expression of gp91(phox). However, DOX treatment significantly increased the expression of gp91 (phox) by 3.0-fold compared with the CON group, and this effect was decreased to 2.1-fold by AOS pretreatment (200 mg/kg/day, seven days). Similar results were found in assessing the expression of lipid peroxide 4-HNE (Figure 5B). AOS by itself had no marked effect on lipid peroxidation compared with the CON group but obviously reduced the accumulation of 4-HNE induced by DOX. The levels of the lipid peroxide 4-HNE in the CON + DOX group and AOS + DOX group were found to be increased by 3.3- and 1.7-fold, respectively, compared to that in the CON group. Representative western blot images are shown in Figure 5C.

### 2.5. AOS Pretreatment Ameliorates DOX-Induced Endoplasmic Reticulum-Mediated Apoptosis in Mice

**Figure 6:**
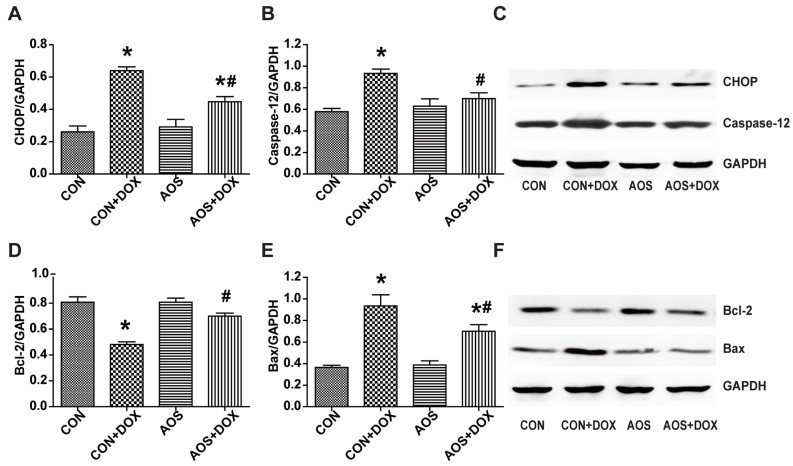
AOS pretreatment inhibits endoplasmic reticulum-mediated apoptosis in hearts treated with DOX. (**A**,**B**) Quantitative analyses of CHOP and Caspase-12 protein expression; (**C**) Representative blots of CHOP, Caspase-12 and GAPDH; (**D**,**E**) Quantitative analyses of Bcl-2 and Bax protein expression; (**F**) Representative blots of Bcl-2, Bax and GAPDH. * *p* < 0.05 vs. the CON group; ^#^ *p* < 0.05 vs. the CON + DOX group, *n* = 6 in each group.

CHOP and Caspase-12 are known to be involved in endoplasmic reticulum-mediated apoptosis in the context of DOX cardiotoxicity [16,17]. Our results demonstrated that AOS by itself had no significant effect on the expressions of CHOP and Caspase-12 compared to the CON group (Figure 6A,B). CHOP and Caspase-12 protein expressions in the CON + DOX group were significantly higher than that of the CON group, and this increase was markedly abolished by AOS pretreatment (200 mg/kg/day, seven days). DOX administration significantly inhibited the expression of Bcl-2 compared to the CON group, whereas AOS pretreatment restored the expression of Bcl-2 to the normal level (Figure 6D). Bax protein expression in the CON + DOX group was significantly higher than that of the CON group, whereas AOS pretreatment significantly decreased the expression (Figure 6E). Representative western blot images are shown in Figure 6C,F.

## 3. Discussion

Treatment progression has improved the survival rate of patients with cancers. However, the progressive, irreversible chemotherapy-induced cardiotoxicity leaves the survivors at a higher risk of death than the general population [18,19]. DOX, one of the most effective chemotherapy agents, is broadly used as one component of treatment protocols, yet its administration is greatly compromised by its cardiotoxicity. Hence, it is urgent for cardio-oncologists to identify strategies that can protect against DOX-induced cardiotoxicity without affecting its anticancer activities. The current study demonstrated that AOS was cardioprotective in the face of acute DOX cardiotoxicity. We found that inhibitions of oxidative stress and endoplasmic reticulum-mediated apoptosis were associated with the cardioprotection.

This present study was the first to demonstrate that mice pre-treated with AOS were well protected against acute DOX cardiotoxicity, as evidenced by a significantly improved survival rate, heart weight to body weight ratio, cardiac contractile dysfunction, histological alterations, and decreased the myocardial apoptosis. Plasma cTnI monitoring is broadly used in clinical practice and animal experiments as a specific biomarker of heart injury [20,21]. The plasma level of cTnI was significantly elevated in the DOX-treated mice, whereas AOS pretreatment down-regulated this increase, indicating the strong cardioprotective efficacy against acute DOX cardiotoxicity. AOS has also been shown to provide protection against many diseases including Alzheimer’s disease and asthma [22,23]. By using neuron-like PC12 cells, Tusi et al. previously showed that AOS decreased H2O2-induced oxidative stress and endoplasmic reticulum-mediated apoptosis [12]. In contrast, AOS was also found to have the capacity to trigger an oxidative burst and induce resistance against infection in a few members of brown algae [24], which reminds us that AOS maybe exhibit different bioactivity in face of different injuries.

Available laboratory evidence suggests that increased oxidative stress is well known as the causative factor in acute DOX cardiotoxicity [5,6,8]. Previous studies have confirmed that DOX induces the overproduction of a cascade of reactive oxygen species (ROS) such as hydroxyl radicals, oxyanions and hydrogen peroxide, which are involved in DOX-induced cardiotoxicity [25,26]. NADPH oxidases, with the primary function of ROS generation, are activated by various stimuli that are important in cardiac remodeling and are also capable of modulating other ROS sources [27]. Using rat embryonic myocyte H9C2 cell cultures, Gilleron et al. reported that the activity of NADPH oxidases increased more than two-fold after one hour of DOX incubation [28]. As a multi-component enzyme, NADPH oxidases are the primary source of oxidative stress in acute DOX cardiotoxicity. Once stimulated, the cytosolic complex migrates to the membrane and assembles with other subunits to form an active oxidase with the ability to produce superoxide anions [29]. The previous study demonstrated that DOX-induced NADPH-dependent superoxide generation was critically dependent on NOX2 (also named gp91 (phox)). Knockout of NOX2 protected the mice against heart failure, cardiomyocyte atrophy, and apoptosis after DOX treatment [8]. Overproduction of oxidants and electrophiles during DOX treatment activates the oxidative response of membrane lipids, leading to the accumulation of 4-HNE, which has been shown to affect mitochondrial function, modify protein functions and be a specific biomarker of DOX-induced oxidative stress [30,31]. Consistent with these studies, the expression of gp91 (phox), the pivotal NADPH oxidase subunit, was up-regulated after DOX administration. Similarly, the expression of the lipid peroxide 4-HNE was also up-regulated. However, pretreatment with AOS inhibited the oxidative stress process by down-regulating the expressions of gp91 (phox) and 4-HNE in the heart. Moreover, our results showed that AOS pretreatment had no obvious effect on the oxidative stress in the heart under physiological conditions.

Endoplasmic reticulum-mediated apoptotic signaling has been studied in the development of heart failure [32,33]. It is also believed that increased oxidative stress can directly induce endoplasmic reticulum stress [34,35]. Chua et al. found that DOX could activate the endoplasmic reticulum-initiated apoptotic response and further augment endoplasmic reticulum stress in mouse hearts [36]. Signal transduction pathways such as the Caspase-12 and C/EBP homologous protein (CHOP) pathways can mediate and augment endoplasmic reticulum-mediated apoptosis [37]. In the context of endoplasmic reticulum stress, Caspase-12, serving as an endoplasmic reticulum membrane-resident pro-apoptotic molecule, activates the caspase cascades which mainly induce cell death [38]. Consistent with previous research that demonstrates that the inhibition of Caspase-12 cleavage is linked with the recovery of heart function [39], our data suggested that AOS pretreatment attenuated Caspase-12 cleavage in the face of DOX insult, leading to decreased myocardial apoptosis and improved cardiac dysfunction. Many studies have demonstrated that CHOP, a marker of endoplasmic reticulum-mediated apoptosis [40], can promote apoptosis by inhibiting expression of the anti-apoptotic factor Bcl-2 and activating expression of the pro-apoptotic factor Bax [41]. Over-expression of CHOP decreased the expression of Bcl-2 protein and facilitated translocation of the Bax protein from the cytosol to the mitochondria [42]. Our data also supported this hypothesis. Administration of DOX significantly activated the CHOP signaling pathway-mediated myocardial apoptosis by regulating the expressions of Bcl-2 and Bax. However, the detrimental effect was ameliorated by AOS pretreatment. Importantly, our results showed that AOS pretreatment had no marked effects on the endoplasmic reticulum mediated-apoptotic signaling pathway under physiological conditions.

## 4. Materials and Methods

### 4.1. Preparation of AOS

**Figure 7:**
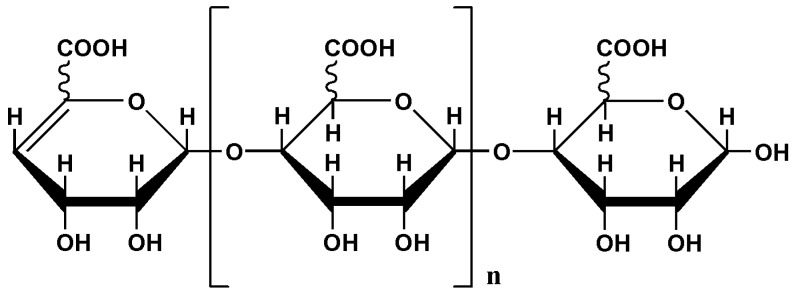
Schematic representation of the molecular structure of alginate oligosaccharide (AOS) prepared by enzymatic degradation.

AOS, a gift from Qingdao BZ Oligo Biotech Co. Ltd (Qingdao, China), was produced by the method of enzymatic degradation as previously described [43]. Briefly, alginate (5 g) derived from *Laminaria japonica* with an M/G ratio of 1.86/1 [44] was dissolved in 500 mL of 50 mmol/L Tris-HCL buffer (pH 7.0), which was added to 200 units of alginate lyase purified from *Pseudomonas* sp. HZJ 216. The enzymatic reaction was performed at 30 °C for 6 h, and then terminated by boiling in water for 5 min. The AOS was initially separated by adjusting the pH of the supernatant to 2.85. Next, the methods of anion-exchange chromatography and desalting were used to further purify the AOS. Then, the methods of High Performance Gel Permeation Chromatography (HPGPC) (Dionex, Sunnyvale, CA, USA) and Electrospray Ionization Mass Spectroscopy (ESI-MS) (Agilent Technologies, Santa Clara, CA, USA) were used to determine the relative molecular mass and degree of polymerization (DP) of the AOS. The relative molecular weight of AOS is approximately 1.2 kDa (Figure S1), and the DP of AOS mainly ranges from 2 to 6 (Figures S2 and S3, Tables S1 and S2). Lastly, the 1H Nuclear Magnetic Resonance (1H-NMR) spectroscopy method was performed to determine the M/G ratio of AOS which is 1/2.6 (Figure S4). The chemical structure of AOS acquired through enzymatic degeneration was shown in Figure 7.

### 4.2. Animals and Drug Administration

Adult male C57BL/6 mice (22–25 g; Department of Laboratory Animal Science, Fudan University, (Shanghai, China) were maintained under specific pathogen-free conditions in an animal room on a 12/12 h light/dark cycle with free access to water and food. All experiments were performed in strict accordance with the guidelines of the China Council on Animal Management. The protocols were approved by the Committee on the Ethics of Animal Experiments of Fudan University. The model of acute DOX cardiotoxicity was induced by intraperitoneal injection of a single dose (15 mg/kg) of DOX (Sigma-Aldrich, St. Louis, MO, USA) [15]. The AOS was dissolved in normal saline with a concentration of 2.0% *w*/*v*. In part one, the mice were randomly divided into the following four groups according to treatment: vehicle control group (CON, *n* = 6), DOX group (CON + DOX, *n* = 6), AOS treatment group (AOS-T, *n* = 6) and AOS treatment + DOX group (AOS-T + DOX, *n* = 6). The mice were treated with AOS (200 mg/kg/day) or an equal volume of normal saline by gavage for 5 consecutive days from the day of DOX injection to the end of the study based on a previous study [21]. In part two, the mice were randomly divided into the following four groups according to treatment: vehicle control group (CON, *n* = 6), DOX group (CON + DOX, *n* = 6), AOS pretreatment group (AOS, *n* = 6), and AOS pretreatment + DOX group (AOS + DOX, *n* = 6). The mice were pretreated with AOS (200 mg/kg/day) or an equal volume of normal saline by gavage for 7 consecutive days, and on the eighth day, the mice were acutely injected intraperitoneally with DOX or an equal volume of normal saline. Next, the mice received the same daily dose of AOS (200 mg/kg/day) and normal saline by gavage for another five days. At the end of the study, cardiac function was measured. Following anesthesia, body weights were recorded, blood samples were collected, and the hearts were rapidly removed, weighed, and then snap frozen in liquid nitrogen for further examinations. For survival observation, the mice were treated with a single high dose (20 mg/kg) of DOX, and the mortality was monitored (*n* = 10 in each group) for 15 days after DOX injection. A total of 112 animals were used in the experiments.

### 4.3. Determination of Cardiac Troponin-I Level

On the fifth day after DOX injection, blood samples were collected from the carotid artery with EDTA-containing syringes prior to heart harvest. The plasma was prepared by centrifugation at 4000 rpm for 30 min and 4 °C immediately frozen and stored at −80 °C. The plasma was thawed only once for cardiac troponin-I (cTnI) measurement. The level of plasma cTnI was quantified with a Mouse Cardiac troponin-I for plasma kit (Nanjing Jiancheng Bioengineering Institute, Nanjing, China). The assay was implemented according to the protocol provided by the manufacturer.

### 4.4. Hemodynamic Measurements

Transthoracic echocardiography was performed by using an animal specific instrument (Vevo707B, Visual Sonics Inc., Toronto, ON, Canada) on the fifth day after DOX and normal saline injection. The mice were anesthetized with 1%–2% isoflurane, and the heart rates were stably maintained between 450 and 500 beats per min. After the B- and M-mode images were acquired, the left ventricular end-diastolic dimension (LVEDD), left ventricular end-systolic dimension (LVESD), left ventricular ejection fraction (LVEF) and left ventricular fractional shortening (LVFS) were measured as previously described [45]. All measurements were performed by three experienced technicians who were blinded to the animal groups.

### 4.5. Histopathological Analysis

The heart tissue was immediately placed in 10% neutral buffered formalin for 24 h. Then, the specimen was embedded in paraffin, and serial sections were cut at 4 μm thickness. The sections were stained with hematoxylin and eosin (HE). Eight microscopic fields (400×) from each section were captured from each slide and imaged with a color digital camera.

### 4.6. Detection of Myocardial Apoptosis

Myocardial apoptosis was examined by using a terminal deoxynucleotidyl transferase-mediated dUTP nick end labeling (TUNEL) method. Paraffin-embedded sections of heart tissues were stained using an In Situ Cell Apoptosis Detection Kit (POD; Roche Diagnostics Corp, Indianapolis, IN, USA) according to the manufacturer’s protocol. Eight microscopic fields (400×) from each section were analyzed by counting brown nuclei. The percentage of TUNEL-positive nuclei (brown nuclei) was calculated.

### 4.7. Western Blot Analyses

Hearts were quickly removed and frozen in liquid nitrogen. Total protein was extracted from the homogenized heart tissues using RIPA lysis buffer (Beyotime Biotechnology, Nanjing, China) and a complete protease inhibitor cocktail. Subsequently, extracted proteins (50 μg) were loaded in a 10% or 12% polyacrylamide gel and then transferred to a polyvinylidene fluoride membrane. The protein expression was detected by immunoblotting with antibodies against gp91 (phox), 4-HEN, Caspase-12 (Abcam), CHOP, Bax, and Bcl-2 (Cell Signaling Technology). After three washes, the blots were incubated with radish peroxidase-conjugated rabbit secondary antibody immunoglobulin G (Kangchen Biotechnology, Shanghai, China). Glyceraldehyde-3-phosphate dehydrogenase (GAPDH) served as the loading control. The protein bands were detected by chemiluminescence and quantified using a Bio-Rad equipped with image software basic Quantity One (Bio-Rad, Hercules, CA, USA).

### 4.8. Statistical Analysis

Data were expressed as the mean ± standard deviation and compared by one-way analysis of variance (ANOVA) followed by a Student-Newman-Keuls (SNK) post hoc test using Graph Pad Prism 6 software (San Diego, CA, SA). Survival curves were determined by the Kaplan-Meier estimator and compared by a log-rank test. A *p* < 0.05 was considered statistically significant.

## 5. Conclusions

The current study is the first to demonstrate that AOS successfully prevents acute DOX cardiotoxicity in mice, at least in part, by suppression of oxidative stress and endoplasmic reticulum-mediated apoptosis. Our data indicate that AOS may clinically serve as a novel preventive strategy against acute DOX cardiotoxicity. However, we should not ignore the question of whether AOS administration interferes with the anticancer activity of DOX, which was not illuminated in the present study. Moreover, the pharmacokinetics of AOS were also not investigated. The challenge for future studies is to focus on answering these important questions.

## Supplementary Materials

The following are available online at www.mdpi.com/1660-3397/14/12/231/s1, Figure S1: The relative molecular mass of AOS analyzed by High Performance Gel Permeation Chromatography (HPGPC). The relative molecular mass of AOS is 1.2 kDa, which was determined by the different molecular weights of dextran standards from the National Institutes for Food and Drug Control, Figure S2: The degree of polymerization (DP) of AOS analyzed by Electrospray Ionization Mass Spectroscopy (ESI-MS), Figure S3: The different degree of polymerization (DP) of AOS analyzed by High Performance Gel Permeation Chromatography (HPGPC), Figure S4: The M/G ratio of AOS analyzed by 1H Nuclear Magnetic Resonance (1H-NMR) spectroscopy. The M/G ratio of AOS was approximately 1/2.6 by calculating the integral areas of the M and G residues, Figure S5: Effects of AOS administered after DOX injection on cardiac dysfunction induced by DOX insult. (A) The cardiac function of mice receiving DOX injection with or without AOS (200 mg/kg, 5 days) treatment was measured after 5 days, and representative echocardiographic images were acquired; (B–E) Data of cardiac function were collected. LVEDD: left ventricular end-diastolic dimension; LVESD: left ventricular end-systolic dimension; EF: left ventricular ejection fraction; and FS: left ventricular fractional shortening. * *p* < 0.05 vs. the CON group; *n* = 6 in each group, Table S1: Electrospray Ionization Mass Spectroscopy (ESI-MS) data analysis of AOS. The DP of AOS mainly ranged from 2 to 6, Table S2: The relative percentages of the different degrees of polymerization of AOS. The AOS mainly contains DP2~DP6; the relative contents of different DP are DP2 26.27%, DP3 32.50%, DP4 18.55%, DP5 13.98%, DP6 3.69%, and >DP7 5.01%, according to the peak area normalization method.
