# Supplementary material for: Alginate Oligosaccharide Prevents Acute Doxorubicin Cardiotoxicity by Suppressing Oxidative Stress and Endoplasmic Reticulum-Mediated Apoptosis
Source: Mar Drugs. 2016 Dec 20;14(12):231. doi: 10.3390/md14120231 (PMC5192468; doi:10.3390/md14120231)
Supplement: Supplementary file 1 [file marinedrugs-14-00231-s001.docx]

**Supplementary Materials: Alginate Oligosaccharide Prevents Acute Doxorubicin Cardiotoxicity by Suppressing Oxidative Stress and Endoplasmic Reticulum-Mediated Apoptosis**

Jun-Jie Guo, Lei-Lei Ma, Hong-Tao Shi, Jian-Bing Zhu, Jian Wu, Zhi-Wen Ding, Yi An,
Yun-Zeng Zou and Jun-Bo Ge


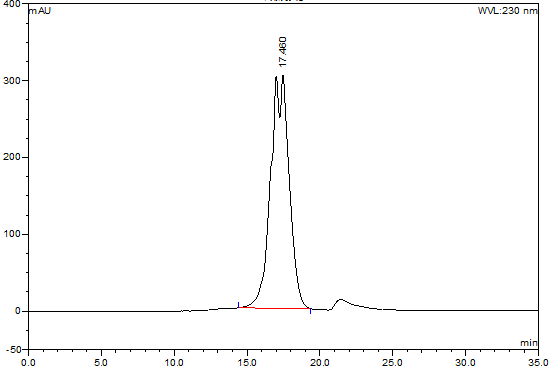


**Figure S1.** The relative molecular mass of AOS analyzed by High Performance Gel Permeation Chromatography (HPGPC). The relative molecular mass of AOS is 1.2 kDa, which was determined by the different molecular weights of dextran standards from the National Institutes for Food and Drug Control.

**Figure S2.** The degree of polymerization (DP) of AOS analyzed by Electrospray Ionization Mass Spectroscopy (ESI-MS).

**Table S1.** Electrospray Ionization Mass Spectroscopy (ESI-MS) data analysis of AOS. The DP of AOS mainly ranged from 2 to 6.

| **Observed Ion (*m*/*z*)** | **Assignment Ion** | ***M*w (Theoretical)** |
| --- | --- | --- |
| 372.9 | [AOS 2+Na-2H]^-^ | 372.1 |
| 571.0 | [AOS 3+2Na-3H]^-^ | 571.1 |
| 769.0 | [AOS 4+3Na-4H]^-^ | 769.1 |
| 967.0 | [AOS 5+4Na-5H]^-^ | 967.2 |
| 1165.0 | [AOS 6+5Na-6H]^-^ | 1165.2 |


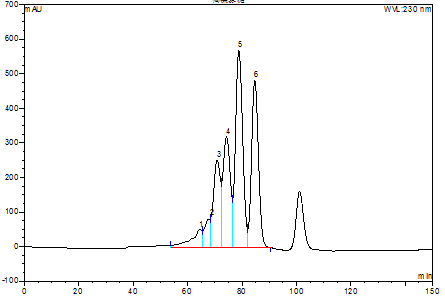


**Figure S3.** The different degree of polymerization (DP) of AOS analyzed by High Performance Gel Permeation Chromatography (HPGPC).

**Table S2.** The relative percentages of the different degrees of polymerization of AOS. The AOS mainly contains DP2~DP6; the relative contents of different DP are DP2 26.27%, DP3 32.50%, DP4 18.55%, DP5 13.98%, DP6 3.69%, and >DP7 5.01%, according to the peak area normalization method.

| **No.** | **Ret. Time (min)** | **Area μRIU × min** | **Rel. Area %** |
| --- | --- | --- | --- |
| 1. >DP7 | 64.46 | 271.442 | 5.01 |
| 2. DP6 | 68.42 | 200.03 | 3.69 |
| 3. DP5 | 70.81 | 757.498 | 13.98 |
| 4. DP4 | 74.26 | 1005.459 | 18.55 |
| 5. DP3 | 78.80 | 1761.316 | 32.50 |
| 6. DP2 | 84.66 | 1423.956 | 26.27 |

*
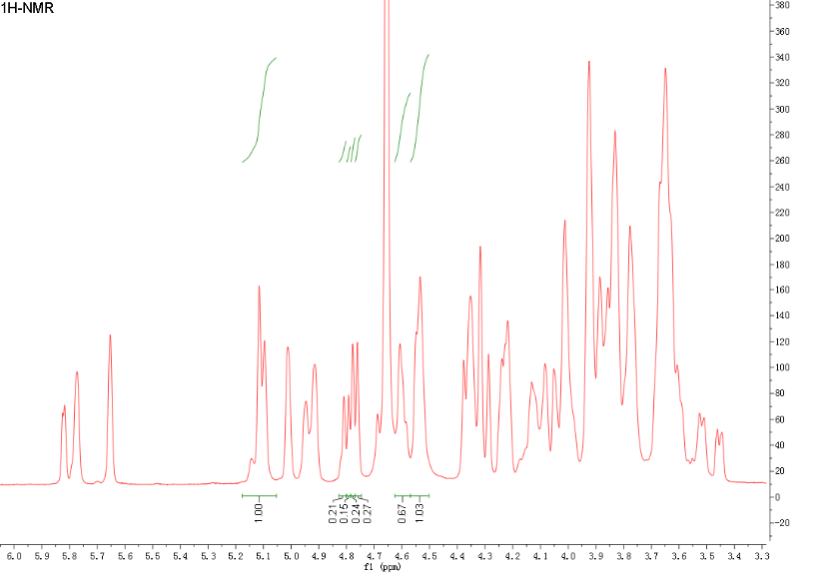
*

**Figure S4.** The M/G ratio of AOS analyzed by 1H Nuclear Magnetic Resonance (1H-NMR) spectroscopy. The M/G ratio of AOS was approximately 1/2.6 by calculating the integral areas of the M and G residues.

*
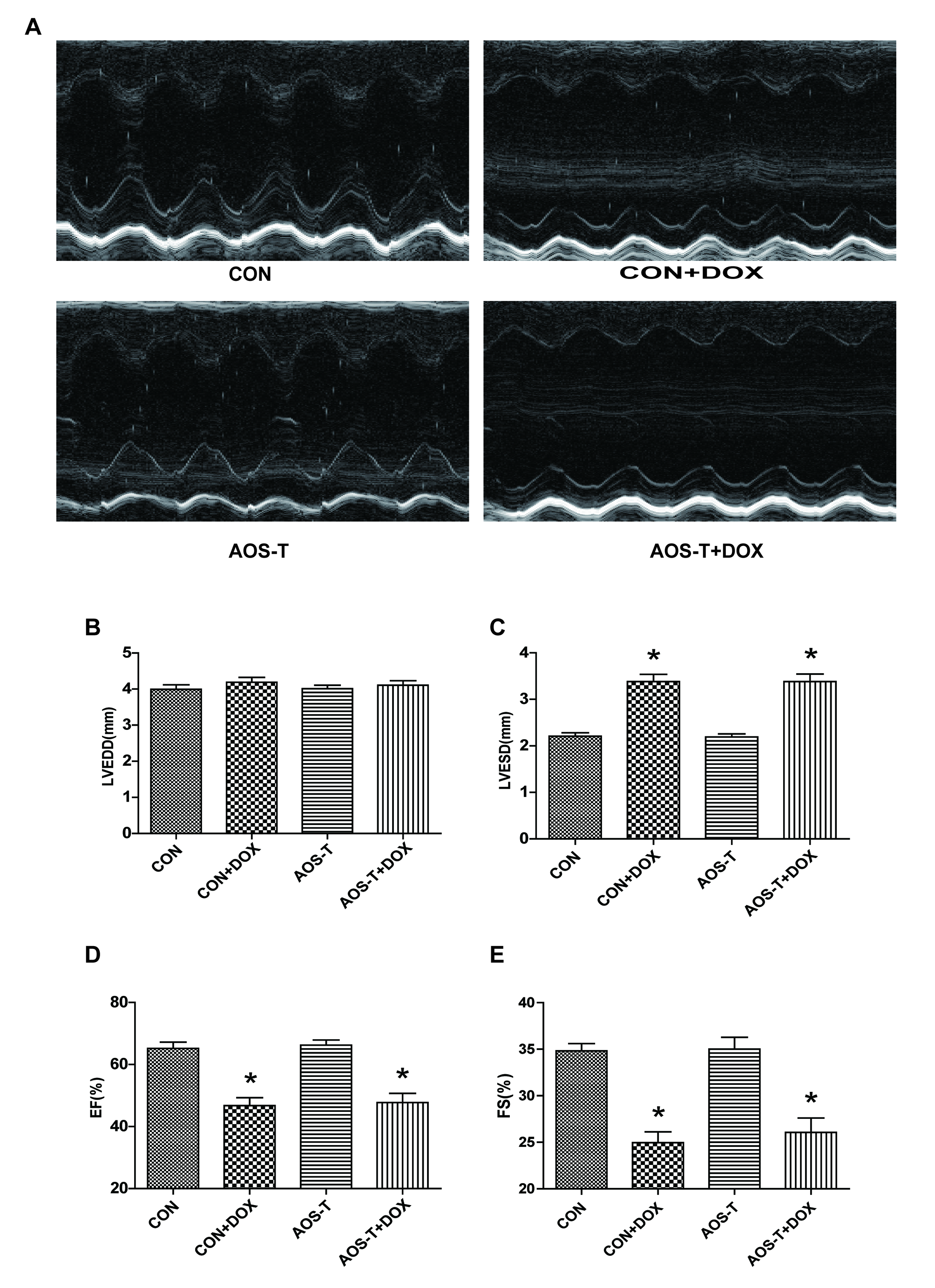
*

**Figure S5.** Effects of AOS administered after DOX injection on cardiac dysfunction induced by DOX insult. (**A**) The cardiac function of mice receiving DOX injection with or without AOS (200 mg/kg, 5 days) treatment was measured after 5 days, and representative echocardiographic images were acquired; (**B**–**E**) Data of cardiac function were collected. LVEDD: left ventricular end-diastolic dimension; LVESD: left ventricular end-systolic dimension; EF: left ventricular ejection fraction; and FS: left ventricular fractional shortening. * *P* < 0.05 vs. the CON group; *n* = 6 in each group.
